# Intracellular Ca^2+^ Signaling and Calcium Release-Activated Calcium Modulator ORAI1 Are Associated With CD4^+^ T Lymphocytes in Dairy Cows

**DOI:** 10.3389/fimmu.2022.835936

**Published:** 2022-05-18

**Authors:** Ming Li, Bingbing Zhang, Yufeng Yin, Jianan Wen, Jingjing Wang, Yuxin He, Qianming Jiang, Juan J. Loor, Shuang Wang, Wei Yang, Chuang Xu

**Affiliations:** ^1^ College of Animal Science and Veterinary Medicine, Heilongjiang Bayi Agricultural University, Daqing, China; ^2^ College of Life Science and Technology, Heilongjiang Bayi Agricultural University, Daqing, China; ^3^ Mammalian NutriPhysio Genomics, Department of Animal Sciences and Division of Nutritional Sciences, University of Illinois, Urbana, IL, United States

**Keywords:** SOCE, fatty acids, CD4^+^ T cells, ketosis, ORAI1

## Abstract

The nutritional status of dairy cows and the metabolism of specific nutrients are critical regulators of immune cell function. Around the time of parturition, mobilization of body lipid and muscle helps compensate for the decrease in nutrient intake and the increased requirements of the mammary gland for lactation. An end-result of these processes is the marked increase in circulating concentrations of fatty acids (FA), which are a major risk factor for immune dysfunction. In food animal species such as dairy cows, any disturbance in nutritional or immunological homeostasis leads to deleterious feedback loops that can further risk health, efficiency of nutrient use, and compromise availability of safe and nutritious dairy foods for humans. Despite substantial progress with respect to regulation of innate immunity, such knowledge for adaptive immunity is scarce. To help bridge this gap in knowledge, we sought to study the role of calcium release-activated calcium modulator ORAI1 activation in T cells systemic immune function *in vivo*. CD4^+^ T cells were isolated from peripheral blood of dairy cows diagnosed as healthy or with ketosis, a common metabolic disorder of FA metabolism. Results revealed that levels of intracellular Ca^2+^ and reactive oxygen species (ROS) along with the abundance of store-operated Ca^2+^ entry (SOCE) moiety increased during ketosis. Further, plasma concentrations of inflammatory cytokines were elevated, the balance of Th17/Treg cells was disrupted, mitochondrial function impaired, and the abundance of mitophagy-related proteins in CD4^+^ T cells altered during ketosis. Molecular characterization of the direct effects of FA was evaluated in CD4^+^ T cells isolated from the spleen of 1-day-old calves. Enhanced supply of FA increased intracellular Ca^2+^ and ROS concentrations, upregulated the abundance of proteins associated with mitochondrial dynamics and ORAI1. Intermediates of mitophagy accumulated and the balance of Th17/Treg cells also was affected by the supply of FA. These negative effects were attenuated by silencing or inhibition of ORAI1 in CD4^+^ T cells. Together, data indicated that physiological states that lead to increases in systemic concentrations of FA could impact adaptive immunity negatively through ORAI1 regulated intracellular Ca^2+^, ROS balance, and increased effector functions of Th17 cells.

## Introduction

Incidence of disease in food-producing animals is a major roadblock to a sustainable animal agriculture sector worldwide. Dairy cows, in particular, are highly-susceptible to both metabolic and infectious diseases around the period of parturition (1). In all mammals, this physiological stage is characterized by mobilization of body fat and muscle reserves that help compensate for the normal and gradual decrease in feed intake as the animal approaches parturition and the onset of lactation (2, 3). Fat stores, in particular, release fatty acids (FA) which travel to the liver where, above a certain threshold, they can cause physiological disturbances that compromise health and production efficiency (4). These adaptations that cows face compromise availability of safe and nutritious dairy foods for humans.

The capacity of pregnant females to prevent the onset of diseases around parturition is closely associated with functioning of the immune system. For instance, lymphocytes recognize bacterial antigens through membrane receptors specific to invading pathogens. T cell activation initiates a transition from quiescence to rapid cell growth, proliferation, and differentiation into functional subsets to drive or suppress the immune response (5). T cells are subsets of lymphocytes, which include CD8^+^ and CD4^+^ (T helper, Th) T cells. Further division of Th cells includes Th1, Th2, Th17, and T regulatory cells (Treg). The balance between pro-inflammatory Th17 cells and anti-inflammatory Treg cells determines the magnitude of immune responses that are critical for homeostasis and health of organisms (6). Mature Th17 cells are characterized by spontaneous production of IL-17A and IL-22, which can induce systemic inflammation (7). Treg cells, which express the transcription factor forkhead box protein P3 (Foxp3) and produce IL-10, help maintain peripheral immune tolerance (8). Similar to neutrophils, increases in circulating FA impair peripheral blood mononuclear cell functions (9) and high concentrations of FA have negative effects on lymphocyte function *in vitro* (10). The *in vivo* mechanisms whereby increases in FA influx into T cells affect their function are unknown. Particularly in the context of the inflammatory response, metabolic disorders, such as ketosis (11), are characterized by high concentrations of circulating FA which occurs most-commonly at the onset of lactation.

Calcium and Ca^2+^-related signaling pathways in endoplasmic reticulum and mitochondria orchestrate crucial events regulating physio- and pathological processes in eukaryotic cells (12). In immune cells, the endoplasmic reticulum Ca^2+^ influx (Store-operated Ca^2+^ entry, or SOCE) machinery, which includes stromal interaction molecule (STIM) and calcium release-activated calcium channel protein ORAI1, is key for cellular calcium homeostasis (13, 14). Channels formed by these two proteins respond to decreased Ca^2+^ levels in the ER lumen and induce Ca^2+^ influx from the extracellular space to the cytosol. In murine CD4^+^ T cells, inhibition of SOCE by gene knockout of ORAI or Stim weakens the function of Th17 cells and ameliorates inflammation (15–17). Furthermore, the amount of Ca^2+^ released from the ER is critical to signal transduction in mitochondria. In fact, mitochondrial Ca^2+^ is an essential determinant of organelle function as an overload of intracellular Ca^2+^ is a pathological signal leading to matrix sensitization to stimuli that induces opening of the mitochondrial permeability transition pore (mPTP). Such events are followed by changes in mitochondrial morphology and initiation of mitophagy.

In addition to Ca^2+^ signaling, mitochondrial oxidative metabolism is a major source of ROS in the cell. In fact, Ca^2+^ concentrations can regulate cellular antioxidant defense systems (18) and lead to oxidative damage, inhibition of the respiratory chain, and further generation of ROS (19). Furthermore, different T-cell subsets also have distinct sensitivity to ROS levels, which may influence their development and function. For instance, ROS could induce Th17 cell activation (20), and a decrease in Th17 cell differentiation due to excessive ROS levels could prevent the onset of autoimmune disorders (21).

The underlying causes of dysfunctional inflammation during the period around parturition have been the subject of considerable research. However, the role of adaptive immune responses especially at the onset of lactation when the liver is flooded by FA that often lead to ketosis is largely unknown. The mechanisms whereby SOCE regulates the development and function of T cells are also poorly understood. Thus, the main objective of the present study was to investigate the link between high concentrations of FA, ORAI1 and Ca^2+^ signaling, mitochondrial dysfunction, and the function of T lymphocytes. Clinical ketosis during the first week of lactation provided a physiologically relevant model to study these aspects. *In vitro* studies with isolated CD4^+^ T cells allowed for the controlled evaluation of mechanisms at play.

## Methods and Materials

The Ethics Committee for the Use and Care of Animals, Heilongjiang Bayi Agricultural University (Daqing, China) approved the animal use protocol.

### Animals

Lactating multiparous Holstein cows were selected from a 1,000-cow dairy farm with free-stall housing systems located in Daqing, Heilongjiang Province, China. We chose lactating Holstein cows with a similar number of lactations (2.53 ± 0.11) and at a similar stage of lactation [11.86 ± 0.44 day in milk (DIM) (Sui, #218)]. Veterinarians classified cows as ketotic if milk yield and a nitroprusside test for ketone bodies in milk was positive (22). Accordingly, we preselected 20 cows suspected of being ketotic and 16 healthy cows. Subsequently, blood concentrations of FA (healthy concentrations: 0.361 ± 0.128 mmol/L), hydroxybutyrate (BHBA, healthy concentrations: BHBA ≤ 1.2 mmol/L), and glucose (healthy concentrations: 2.9 – 5.1 mmol/L, **Table 1**) were measured using commercial kits in an automatic clinical analyzer (Synchron DXC800; Beckman Coulter, Inc., Brea, CA). According to these metrics (23, 24), 6 ketotic cows with serum BHB concentration higher than 1.6 mM and 6 control cows with serum BHB concentration lower than 0.4 mM were randomly selected. The basic description of the cows is shown in **Table 2**. Cows had ad libitum access to the same diet offered twice a day (0530 and 1350 h). All cows were examined to ensure there were no other co-morbidities. Blood samples were collected before feeding (after milking) from the tail vein after sanitizing with iodine scrub and 75% alcohol. Tubes containing sodium heparin anticoagulant (about 50 blood mL from every collection) were used for isolation of CD4^+^ T cells and measurement of cytokines in serum.

**Table 1:** Blood biomarker concentrations in healthy cows and cows with ketosis.

Item	Ketosis (n = 20)		Healthy (n = 16)	
	Median	Interquartile range	Median	Interquartile range
Non-esterified fatty acids (mM)	1.13	1.06, 1.25	0.385	0.323, 0.488
β-hydroxybutyrate (mM)	1.85	1.70, 1.90	0.300	0.200, 0.375
Serum glucose (mM)	2.29	2.16, 2.44	3.47	3.00, 3.60

**Table 2:** Basic performance and energy balance biomarkers in healthy cows and cows with ketosis.

Item	Ketosis (n = 6)		Healthy (n = 6)		P-value
	Median	Interquartile range	Median	Interquartile range	
BW (kg)	650	647, 676	673	667, 680	0.000
Milk production (kg/day)	38.8	37.6, 39.6	36.8	34.7, 38.5	0.579
DMI (kg/d)	17.0	15.6, 18.4	22.3	20.7, 23.1	0.000
Milk production/DMI	2.24	2.10, 2.41	1.66	1.61, 1.75	0.000
Serum glucose (mM)	2.33	2.17, 2.53	3.55	3.18, 3.70	0.107
Non-esterified fatty acids (mM)	1.13	1.06, 1.19	0.330	0.280, 0.438	0.000

### Isolation and Culture of CD4^+^ T Cells

For *in vivo* experiments, we placed the collected peripheral blood at 4°C and returned to the laboratory within 2 hours for cell isolation. Lymphocytes were isolated using a Bovine Peripheral Blood Lymphocyte Separation Kit (Solarbio, Beijing, China) according to the manufacturer’s protocols. After erythrocyte removal, CD4^+^ T cells were selected *via* MACS (MicroBeads sorting) isolation. In brief, lymphocytes were labeled with CD4 Monoclonal antibody (Invitrogen Corporation, China) for 25 min at 4°C, then Anti-Mouse IgG2a+b MicroBeads (Miltenyi Biotec) was used for magnetic labeling for 15 min at 4°C. After labeling, CD4^+^ T cells were separated using MS Columns (Miltenyi Biotec). Subsequently, the column was washed two times with MACS Separation Buffer (Miltenyi Biotec) and CD4^+^ T cells collected through adsorption on the column.

For *in vitro* experiments, 4 healthy female Holstein calves (1 d old, 40-50 kg, fasting, and rectal temperature 38.7 to 39.7°C) from a commercial dairy farm (Daqing, China) were used as spleen donors to isolate CD4^+^ T cells. Briefly, the calves were fasting and immediately transported to the laboratory within three hours after their birth. Subsequently, calves were humanely euthanatized by intravenous thiamylal sodium injection. Fresh spleen parenchyma was harvested aseptically from each calf. The spleen was washed twice with PBS containing 5000 UI/mL Amphotericin and Gentamicin for 10 min, then cut into small pieces before the splenic capsule was removed and ground to obtain splenocytes. The splenocyte suspension was then filtered sequentially with cell sieves of 50 (300 μm), 100 (150 μm), and 200 mesh (75 μm). After erythrocyte removal, the splenocyte suspension was washed twice with RPMI-1640 basic medium (Hyclone Laboratories) and centrifuged for 5 min at 500 × g at 4°C and CD4^+^ T cells positively selected *via* MACS isolation. The labeling method was the same as that for *in vivo* experiments. Cell viability was assessed with the Trypan blue dye (Sigma-Aldrich) exclusion method after isolation. Only cells with viability >95% were used for further experiments. Primary calf CD4^+^ T cells were cultured in 6-well plates at 1 × 10^6^ cells/mL using RPMI-1640 basic medium supplemented with 10% FBS, 1% Penstrep, 1% L-Glut and 55 μM β-mercaptoethanol and 1 μg/mL Concanavalin A for 60 h, and incubated at 37°C in 5% CO_2_.

After 60-h culture, CD4^+^ T cells were maintained in RPMI-1640 basic medium supplemented with 2% BSA and treated with vehicle only or a mixture of 1.2 mM fatty acids for 0, 6, 12, 24, 48, and 72 h. The concentration of fatty acids in the mixture developed was based on previous reports from dairy cows with ketosis (24–26). A stock fatty acid solution was prepared by diluting fatty acid in 0.1 M KOH at 60°C (pH 7.4). The stock fatty acid (52.7 mM) solution included Oleic acid (OA, cis9-18:1, 22.9 mM, 43.5%; Sigma-Aldrich), Linoleic acid (LA, cis9, cis12-18:2, 2.6 mM, 4.93%; SigmaAldrich), Palmitic acid (PA, 16:0, 16.8 mM, 31.9%; Sigma-Aldrich), Stearic acid (SA, 18:0, 7.6 mM, 14.4%; Sigma-Aldrich), and Palmitoleic acid (POA, cis9-16:1, 2.8 mM, 5.31%; Sigma-Aldrich).

### Silencing and Inhibition

For silencing, 1 × 10^6^ CD4^+^ T cells (6-well plate) were seeded 24 h before the experiment in antibiotic-free medium. Cells were transfected with 40 pM cattle ORAI1 siRNA (GenePharma, Shanghai, China) and a non-target siRNA using siRNA-mate (GenePharma, Shanghai, China) according to the manufacturer’s protocols. For inhibition, CD4^+^ T cells were pretreated with 50 mM of the store-operated Ca^2+^ Channel Inhibitor 2APB (MedChemExpress, Shanghai, China) for 2 h prior to fatty acid challenge. Ten mM of the OXS inhibitor N-Acetylcysteine (NAC, Beyotime Biotechnology, Jiangsu, China), or vehicle (DMSO) was used for 2 h prior to fatty acid challenge.

### Measurement of Serum Parameters

Blood samples were collected use tubes containing sodium heparin anticoagulant from the tail vein and immediately centrifuged at 1,400 × g for 10 min, and used for measurement of serum parameters within 2 hours. Serum IL-17, IL-10, TGF-β1, and IL-6 concentrations were determined using commercially available kits according to manufacturers’ instructions (Lengton, Shanghai, China). Two measurements are made for the sample and the standard curve. All ELISA plates were read using a SpectraMax Plus384 microplate reader (Molecular Devices, Downingtown, PA). Samples were run in duplicate, and duplicates with coefficients of variance (CV) less than 10% were included for analysis according to the instructions (average intra-assay CVs: IL-6 = 9.03%; IL-10 = 9.05%; IL-17 = 8.18% and TGF-β = 9.09%). To control for inter-assay variation, a pooled sample was run on each plate and duplicates with coefficients of variance less than 12% were included for analysis according to the instructions (average inter-assay CVs: IL-6 = 6.13%; IL-10 = 7.13%; IL-17 = 9.74% and TGF-β = 8.69%).

### Cell Viability Assay

Cell viability was determined with the Cell Counting Kit-8 (Beyotime Biotechnology, Jiangsu, China) according to manufacturer’s instructions. Cells were seeded at 5 × 10^3^ cells/well in 96-well plates using RPMI-1640 basic medium supplemented with 10% FBS, 1% Penstrep, 1% L-Glut and 55 μM β-mercaptoethanol and 1 μg/mL Concanavalin A for 60 h, and incubated at 37°C in 5% CO_2_. After 60-h culture, cells were maintained in RPMI-1640 basic medium supplemented with 2% BSA and treated with or without 1.2 mM fatty acids for 0, 6, 12, 24, 48, and 72 h. After treatment, 20 µL of CCK-8 solution was added to each well and then incubated in the dark for 1 h at 37°C in 5% CO_2_. The optical density was measured at 450 nm on a spectrophotometer (Thermo Scientific Instruments Inc.). The experiments were repeated at least three times.

### RNA Isolation and qRT-PCR

Total RNA was isolated using TRIzol (Invitrogen Corporation, China) according to the manufacturer’s protocols. RNA was dissolved in UItraPure Distilled Water (DNAse, RNAse, Free). Concentrations of RNA were determined by measuring absorbance at 260 nm and the purity of RNA was determined by calculating the ratio of absorbance at 260 and 280 nm with values ranging from 1.8 to 2.0. The isolated RNA was stored at -80°C. Reverse-transcription was performed with a PrimeScript RT reagent kit containing Gdna Eraser (Takara Bio, Dalian, China) as recommended by the manufacturer. The RNA was diluted to the optimal concentration and tested with qRT-PCR probes and primers. All qRT-PCR reactions were run in triplicate. The reaction system contained 10 μL of SYBR Green Master, 2 μL primers (1 μL forward primer and 1 μL reverse primer), 2 μL of cDNA templates, and 6 μL of RNase-free distilled H_2_O. The temperature program was: denaturation at 95°C for 3 minutes, a total of 40 cycles of amplification (denaturation at 95°C for 15 seconds, annealing at 60°C for 1 and extension at 60°C for 1 min), and a final extension at 72°C for 5 min. Calculated mRNA abundance in each sample was normalized to GAPDH and ACTB. Relative abundance was quantified with the 2^−ΔΔCT^ method. Reactions were performed in a Bio-Rad iCycler iQTM Real-Time PCR Detection System (Bio-Rad Laboratories Inc., Hercules, CA). Primer sequences used for qPCR are listed in **Table 3**.

**Table 3:** Sequences of primers used for real-time PCR amplification.

Gene	Primer (5’ to 3’)	Primer length	Tm	Gene bank accession number
*β-actin*	Forward: GCCCTGAGGCTCTCTTCCA	19	60.99	NM_173979.3
	Reverse: GCGGATGTCGACGTCACA	18	60.13	
*GAPDH*	Forward: GTCTTCACTACCATGGAGAAGG	22	57.87	NM_001034034.2
	Reverse: TCATGGATGACCTTGGCCAG	20	59.74	
*IL-17A*	Forward: CACAGCATGTGAGGGTCAAC	20	59.12	NM_001008412.2
	Reverse: GTGGAGAGTCCAAGGTGAGG	20	59.39	
*STAT3*	Forward: GCGAGAGGGAGAAAGAGGAAACATC	25	63.13	NM_001012671.2
	Reverse: AGGCACTTGACTCACAACGACAAC	24	63.74	
*IL-22*	Forward: CTGTAGGCTCAACGAGTCCG	20	60.18	NM_001098379.1
	Reverse: CGCTTCGTCACCTGATGGAT	20	60.18	
*FOXP3*	Forward: TGGTGCAATCTCTGGAGCAA	20	59.60	XM_005228107.4
	Reverse: GTCAGATGATGCCGCAGATG	20	59.13	
*IL-10*	Forward: CAAGAGCAAGGCGGTGGAGAAG	22	63.68	NM_174088.1
	Reverse: AACTCACTCATGGCTTTGTAGACACC	26	63.13	
*TGF-β*	Forward: CCAGAGTGCCTGAACAACGGATC	23	63.56	XM_024976323.1
	Reverse: TGGAGCCATTCGTGAACAGCATC	23	63.58	
*ORAI1*	Forward: GCCACCACCGAGAGCCAGAG	20	64.97	XM_005217843.4
	Reverse: CACAACTGACGCTGAACGGAGAG	23	63.74	
*ORAI2*	Forward: CACCTGTTCGCCCTGCTCATTAG	23	63.62	NM_001191348.3
	Reverse: CTCGCTGATGGAGTTGAGGTTGTG	24	63.67	
*ORAI3*	Forward: ACACCAGAGACTCCACCGCTAC	22	63.67	NM_001193202.3
	Reverse: CCAGCCAACCAGGACAACTTCAG	23	63.73	
*STIM1*	Forward: TCTCTCACCCACACCTGCTTCTC	23	63.77	XM_024975390.1
	Reverse: AGCCGACTGTCCTGAGCCTTAG	22	63.75	
*STIM2*	Forward: CCCGAGTGTTGCCAGGATAAGC	22	63.51	XM_024993529.1
	Reverse: ACCAAGTCAGCCAAGCCAGTTC	22	63.29	

### Protein Extraction and Western Blots

Total protein was isolated from CD4^+^ T cells using RIPA Lysis and Extraction Buffer (Beyotime Biotechnology, Jiangsu, China) including protease inhibitors, placed on ice for 30 min, and centrifuged at 12,000 × g for 5 min at 4°C. Protein concentrations were measured using the BCA protein assay kit (Beyotime, China).

For western blot, after high temperature denaturation treatment, the same amount (30 μg/lane) of the protein sample was placed on a 10% SDS-PAGE for separation. Then, protein bands were transferred to a polyvinylidene difluoride (PVDF) membrane (Millipore Corp., Billerica, MA). The membrane was then blocked for 1 h at room temperature in blocking buffer (0.1% Triton-X/PBS containing 5% skim milk powder). Blocked membranes were incubated overnight at 4°C with specific antibodies for total OXPHOS (1:1000, ab110413; abcam, CA), DRP1 (1:1000, ab154879; abcam, CA), MFN2 (1:1000, 12186-1-AP; ProteinTech), FIS1 (1:1000, 10956-1-AP; ProteinTech), SQSTM1/P62 (1:1000, 18420-1-AP; ProteinTech), Parkin (1:1000, #4221; Cell Signaling, Shanghai), LC3B (1:1000, ab48394; abcam, CA), Orai1 (1:1000, ab175040; abcam, CA), ORAI3 (1:1000, ab25426; abcam, CA), STIM1 (1:1000, ab108994; abcam, CA), STIM2 (1:1000, ab59342; abcam, CA), and β-actin (internal control; 1:1000, sc-47778; Santa Cruz, CA). Subsequently, membranes were incubated with HRP-conjugated secondary antibodies (3:5000; Beyotime) for 1 h at room temperature. Immunoreactive bands were detected using an enhanced chemiluminescence solution (Beyotime). Visualization of target proteins was performed using a ProteinSimple imager (ProteinSimple, San Jose, CA). Image Lab software (Bio-Rad, Hercules, CA) was used to analyze intensity of the bands and compared changes across the different treatments.

### Flow Cytometry

Cytosolic Ca^2+^ concentrations were analyzed by flow cytometry. Cells were washed and stained with 3 μM Fluo-3AM (Biotinium, China) in Tyrode buffer (pH 7.4), cultured at 37°C for 30 min.

To determine intracellular change in ROS generation in CD4+ T cells, they were stained using a ROS assay kit (Beyotime). A total of 1 × 10^5^ cells were incubated with 10 μM carboxy-2’,7’-dichloro-dihydro-fluorescein diacetate probe to stain intracellular ROS in PBS for 30 min at 37 °C. Fluorescence was measured at 488 nm (excitation) and 525 nm (emission).

For cytokine staining, cells were re-stimulated with 1 μM ionomycin and 50 ng/mL PMA (phorbol myristate acetate) in the presence of 5 μM Brefeldin A (eBioscience) for 4 h. Cell surface staining was performed use CD4 Antibody (Invitrogen) in PBS, followed by permeabilization with Foxp3/Transcription Factor Staining Buffer Set (eBioscience) and intercellular staining in permeabilization buffer (eBioscience). The following antibodies were used: anti-IL-17A (eBio17B7; eBioscience) antibodies and anti-Foxp3 (FJK-16s; Invitrogen) antibody.

### Immunofluorescence Assay

For calcein release, treated cells were transferred to a cell culture plate pre-coated with 1 x polylysine, and cells washed twice with a modified HBSS-buffered (10 mM HEPES, 2 mM l-glutamine, and 100 μM succinate) (14175095, Thermo Fisher Scientific) saline solution preheated at 37°C. Then, samples were incubated with labeling solution (com- posed of 1 mM calcein and 1 mM CoCl_2_) for 15 min protected from light at 37°C. Cells were then washed with warm modified HBSS buffer to remove residual dye. Samples were placed on a shaker at room temperature for 5 min. Opening of the mPTP was assessed *via* fluorescence using a laser confocal microscope (LSM 5 PASCAL, Zeiss, Oberkochen, Germany).

To detect cell mitochondrial ROS (Mito-SOX, Invitrogen) and Ca^2+^ (Rhod-2-AM, Invitrogen), treated cells were adjusted to a density of 1 × 10^6^ and transferred to a cell culture plate pre-coated with 1 × polylysine. Then, cells were placed in a constant temperature incubator under 5% CO_2_, 37°C conditions for 20 min. Cell culture media was then removed and cells washed twice with a modified HBSS-buffered (10 mM HEPES, 2 mM L-glutamine, and 100 μM succinate) (14175095, Thermo Fisher Scientific) saline solution preheated at 37°C for staining. For staining, cells were diluted with Ca^2+^ and Mg^2+^-containing HBSS buffer to a working solution with a final concentration of 5 μM Mito-SOX for ROS and incubated at 37°C for 10 min, or 4 μM Rhod-2-AM for Ca^2+^ for 40 min. Cells were then washed three times with warm modified HBSS buffer to remove residual dye. A laser confocal microscope was used to determine concentrations (LSM 5 PASCAL, Zeiss, Oberkochen, Germany).

### Metabolic Phenotyping

Oxygen consumption rate (OCR) was measured using the Seahorse XFe bioanalyzer. 1 × 10^6^ CD4^+^ T cells per well were spun onto poly-D-lysine coated seahorse 24 well plates and preincubated in Seahorse XF media (non-buffered RMPI + 10 mM L-glutamine + 10 mM sodium pyruvate + 25 mM glucose) at 37°C for a minimum of 30 min in the absence of CO_2_. OCR was measured under basal conditions, and after addition of the following drugs: 1.5 μM oligomycin, 1.0 μM flurorcarbonyl cyanide phenylhydrazone (FCCP) and 500 nM rotenone + 500 nM antimycin A. Measurements were obtained using a 24-well Extracellular Flux Analyzer (Seahorse Bioscience, California, America).

### Statistical Analysis

Baseline characteristics of the dairy cows are reported as the median and interquartile range, and other data as the means ± SEM, with n denoting the number of independent experiments. Statistical analysis was conducted using SPSS 26.0 Software (IBM, Chicago, IL) and GraphPad Prism program (Prism 8.0; GraphPad Software, San Diego, CA). All data were tested for normality and homogeneity of variance using the Shapiro–Wilk and Levene tests. For baseline characteristics of the dairy cows, data with skewed distribution were analyzed using a nonparametric statistical analysis using the Wilcoxon test (24). For other data with Gaussian distribution, parametric statistical analysis was performed using an independent sample t-test for two groups; one-way analysis of variance (ANOVA) was performed for multiple comparisons with Bonferroni correction. Statistical significance was evaluated *via* unpaired Student’s t-test or one-way ANOVA with a Duncan test for *post hoc* analysis. Only differences with P-value ≤ 0.05 were considered statistically significant and a P-value ≤ 0.01 was considered highly significant.

## Results

### CD4^+^ T Cells From Cows With Ketosis Produce a Th17 Profile Preferentially

**Figure 1:**
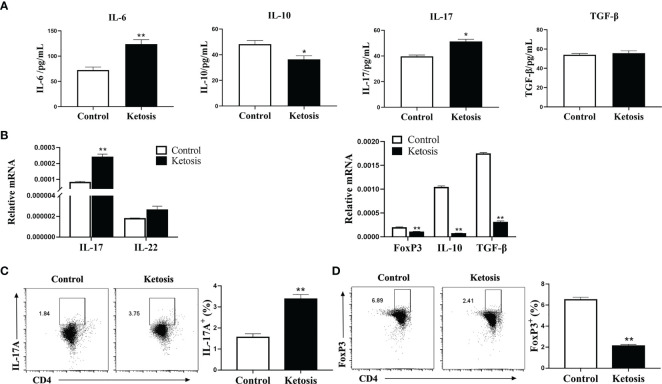
Inflammation increases due to ketosis *in vivo*. **(A)** Levels of IL-17, IL-10, IL-6 and TGF-β in serum from healthy and ketotic cows (n = 6 per group). **(B–D)** CD4^+^ T cells were sorted from healthy and ketotic cows (n = 6 per group). **(B)** Pro-inflammatory and anti-inflammatory factor mRNA expression measured by qRT-PCR **(C)** Populations of IL-17 cytokine-expressing CD4 T cells in peripheral blood from healthy and ketotic cows. **(D)** Populations of FoxP3 expressing CD4^+^ T cells in peripheral blood from healthy and ketotic cows. For all bar plots shown, data are expressed as the means ± SEM, *P < 0.05, **P < 0.01 by independent-samples t-test.

To explore the contribution of ketosis to the CD4^+^ T cells associated with inflammatory diseases, the levels of the inflammatory markers in serum were investigated firstly. Levels of serum IL-17 and IL-6 were greater and IL-10 lower, but TGF-β did not differ in cows with ketosis (**Figure 1A**). Next, CD4^+^ T cells were isolated from blood to test whether ketosis affects CD4^+^ T cells *in vivo*. Results showed that mRNA abundance of IL-17 and IL-22 was upregulated, while Foxp3, IL-10, and TGF-β were downregulated in ketotic cows (**Figure 1B**). The substantial amounts of IL-17A coupled with low amounts of FoxP3 underscored the marked imbalance of Th17/Treg immunity during ketosis (**Figures 1C, D**). These results suggested that ketosis induces Th17/Treg cell immune imbalance, and the body is in a state of inflammation.

### Ca^2+^ Content Is Elevated and SOCE Activated in CD4^+^ T Cells From Ketotic Cows

**Figure 2:**
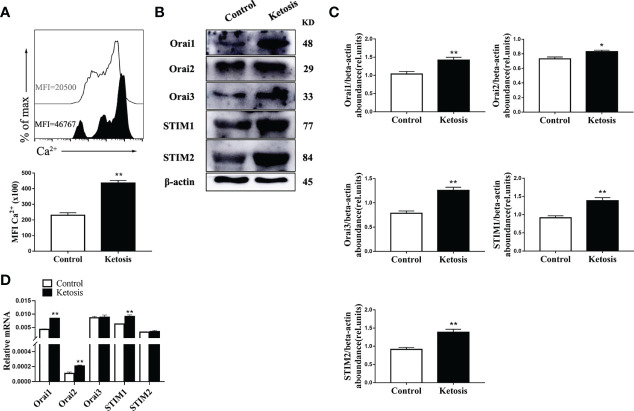
Ketosis increased the levels of Ca^2+^ and expression of SOCE. **(A)** Staining of intracellular Ca^2+^ using Fluo-3A (n = 4 per group). **(B)** Western blot analysis of SOCE-related protein (n = 6 per group) **(C)** Relative protein abundance of SOCE (n = 6 per group). **(D)** ORAI and STIM mRNA expression were measured by qRT-PCR (n = 6 per group). For all bar plots shown, data are expressed as the means ± SEM, *P < 0.05, **P < 0.01 by independent-samples t-test.

To investigate the role of SOCE in ketotic cows CD4^+^ T cells, we measured the Ca^2+^ levels. Flow cytometry revealed that intracellular Ca^2+^ levels were greater in CD4^+^ T cells from ketotic cows (**Figure 2A**). Furthermore, abundance of SOCE composition-associated protein and mRNA of ORAI and STIM were both greater in CD4^+^ T cells from ketotic cows (**Figures 2B–D**). These findings suggested that SOCE is activated in CD4^+^ T cells from ketotic cows.

### Reduced Mitochondrial Function, Increased Levels of ROS, and Initiation of Mitophagy Characterize CD4^+^ T Cells From Ketotic Cows

**Figure 3:**
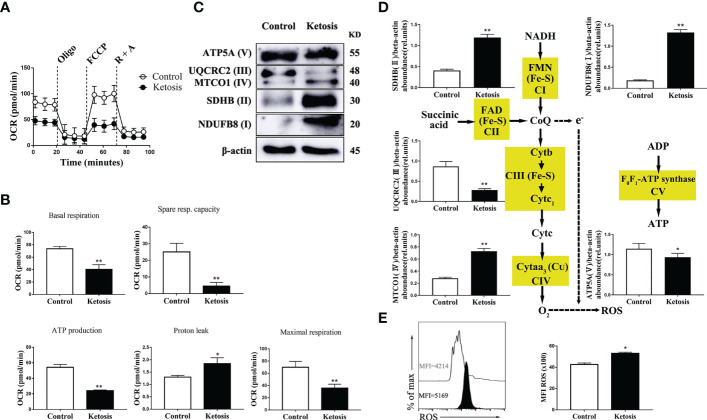
Decreased level of oxidative phosphorylation metabolism following ketosis underlie increased production of ROS in CD4^+^ T cells. **(A–E)** CD4^+^ T cells were sorted from healthy and ketotic cows. **(A)** Oxygen consumption rates (OCRs) measured by extracellular flux analysis (n = 4 per group). T cells were measured under basal conditions. Oligo, oligomycin; R, rotenone; A, antimycin. **(B)** Mean (± SEM) of basal and maximal respiration (after FCCP), ATP production, proton leak and spare respiratory capacity from vivo-isolated CD4^+^ T (n = 4 per group). **(C)** Western blot analysis of ETC complex proteins in CD4^+^ T cells from healthy and ketotic cows (n = 6 per group). **(D)** Relative abundance of ETC complex proteins (n = 6 per group). **(E)** The level of ROS was analyzed by flow cytometry (n = 6 per group). For all bar plots shown, data are expressed as the means ± SEM, *P < 0.05, **P < 0.01 by independent-samples t-test.

To test the mitochondrial function in CD4^+^ T cells from ketotic cows, mitochondrial respiration in CD4^+^ T cells were evaluated. Oxygen consumption rates (OCRs; a readout of mitochondrial respiration) were lower in CD4^+^ T cells from ketotic cows (**Figure 3A**), and there was a greater proton leak and lower basal respiration as well as maximal respiration, spare respiratory capacity (SRC), and ATP production in cells after uncoupling of mitochondria with FCCP (**Figure 3B**). Ketosis was also associated with a significant reduction of protein abundance of complex III (UQCRC2), IV (MTCO1) and V (ATP5A) (**Figures 3C, D**) which explained the defects of mitochondrial respiration. Interestingly, there was increased abundance of complex I (NDUFB8) and II (SDHB) (**Figures 3C, D**) in ketotic cows. Furthermore, CD4^+^ T cells from ketotic cows had greater levels of ROS (**Figure 3E**). Together, this finding is likely explained by the increase of complex I (NDUFB8) and II (SDHB) leading to the increased proton leak of mitochondria.

**Figure 4:**
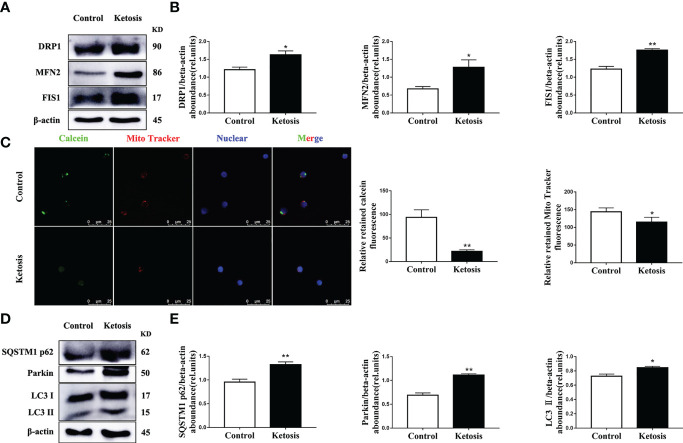
Increased abundance of mitochondrial dynamics-related protein and autophagy-related protein, and impaired mitochondrial membrane permeability. **(A)** Western blot analysis of mitochondrial dynamics-related protein in CD4^+^ T cells from healthy and ketotic cows (n = 6 per group). **(B)** Relative abundance of mitochondrial dynamics-related protein (n = 6 per group). **(C)** Opening of mPTP was analyzed in healthy and ketotic cows (n = 3 per group; 3 random fields per group; scale bars, 25 μm). **(D)** Western blot analysis of autophagy-related protein in CD4^+^ T cells from healthy and ketotic cows (n = 6 per group). **(E)** Relative abundance of autophagy-related protein (n = 6 per group). For all bar plots shown, data are expressed as the means ± SEM, *P < 0.05, **P < 0.01 by independent-samples t-test.

In addition, the abundance of proteins associated with mitochondrial dynamics was upregulated (**Figures 4A, B**) and mitochondrial membrane permeability downregulated (**Figure 4C**) in CD4^+^ T cells from ketotic cows. In terms of mitophagy, the protein abundance of LC3, p62 and Parkin was upregulated in CD4^+^ T cells from ketotic cows (**Figures 4D, E**). Together, these findings demonstrated that mitochondrial protein expression and function were impaired, and mitophagy was initiated in CD4^+^ T cells from ketotic cows.

### Exogenous Fatty Acids Affect Cell Proliferation and mRNA Abundance of Th17-Related Factors

**Figure 5:**
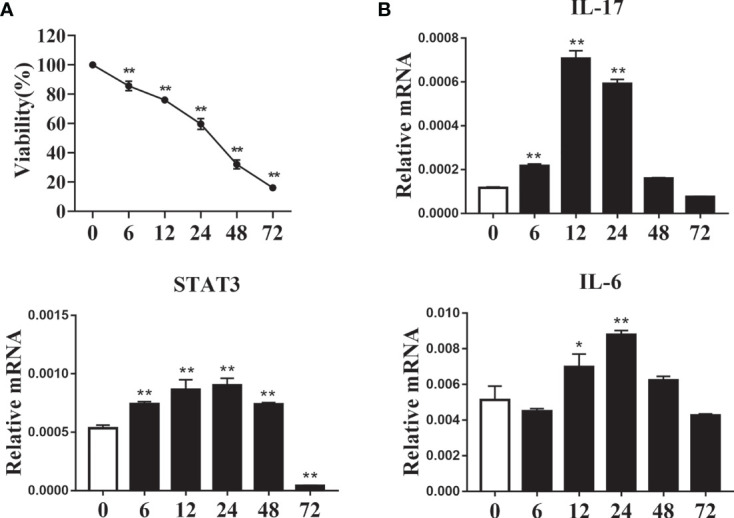
Exogenous fatty acids induced cell damage and increased expression of Th17 cells related factor. CD4^+^ T cells were sorted from spleens from 1-d old calves and treated with 1.2 mM fatty acids for 0, 6, 12, 24, 48 and 72 h **(A)** Cell viability was detected by CCK-8 kit (n = 4 per group). **(B)** Relative mRNA expression of IL-17, STAT3 and IL-6 (n = 4 per group). Comparisons among groups were calculated using a one-way ANOVA with a Duncan correction. The data presented are the mean ± SEM, *P ≤ 0.05, **P ≤ 0.01.

We simulated the effect of increased fatty acid content in the blood of ketotic cows on CD4^+^ T cells. CD4^+^ T cells were from 1 d old calves and stimulated with 1.2 mM fatty acid for different times *in vitro*. Following treatment with fatty acids, the level of cell proliferation decreased gradually (**Figure 5A**). The mRNA abundance of IL-17 reached a peak at 12 h post-fatty acid treatment, and STAT3 had a similar trend (**Figure 5B**). Furthermore, mRNA abundance of IL-6 increased gradually in a time-dependent manner, reaching a peak at 24 h post fatty acid treatment (**Figure 5B**). These results suggested that exogenous fatty acid increased the expression of Th17-related cell factors in CD4^+^ T cells.

### ORAI1 Controls ROS and Ca^2+^ Levels in CD4 T Cells

**Figure 6:**
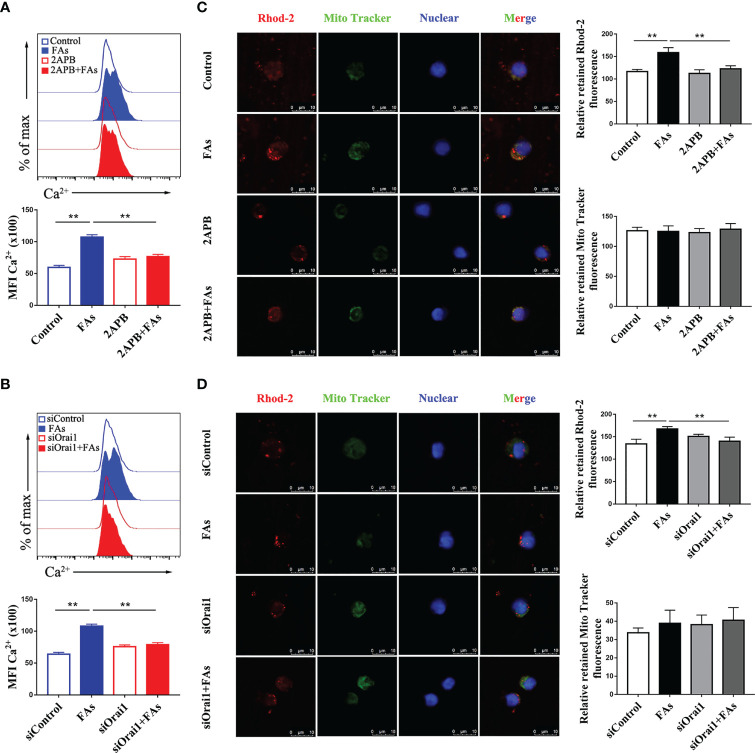
Inhibition or silencing of ORAI1 relieved FAs-induce increased level of intracellular Ca^2+^ and mitochondrial Ca^2+^ in CD4^+^ T cells. CD4^+^ T cells were sorted from spleens from 1-d old calves and stimulated with 2APB or siORAI1 before FAs treatment with 12 h **(A)** Staining of intracellular Ca^2+^ use Fluo-3A in stimulated with 2APB (n = 4 per group). **(B)** Staining of intracellular Ca^2+^ use Fluo-3A in stimulated with siORAI1 (n = 4 per group). **(C)** Staining of mitochondrial Ca^2+^ use Rhod-2 in stimulated with 2APB (n = 3 per group; 3 random fields per group; scale bars, 10 μm). **(D)** Staining of mitochondrial Ca^2+^ use Rhod-2 in stimulated with siORAI1(n = 3 per group; 3 random fields per group; scale bars, 10 μm). The data of the control were used to normalize other treatments. Comparisons among groups were calculated using a one-way ANOVA with a Duncan correction. The data presented are the mean ± SEM, **P ≤ 0.01 indicate differences from control or FAs alone.

**Figure 7:**
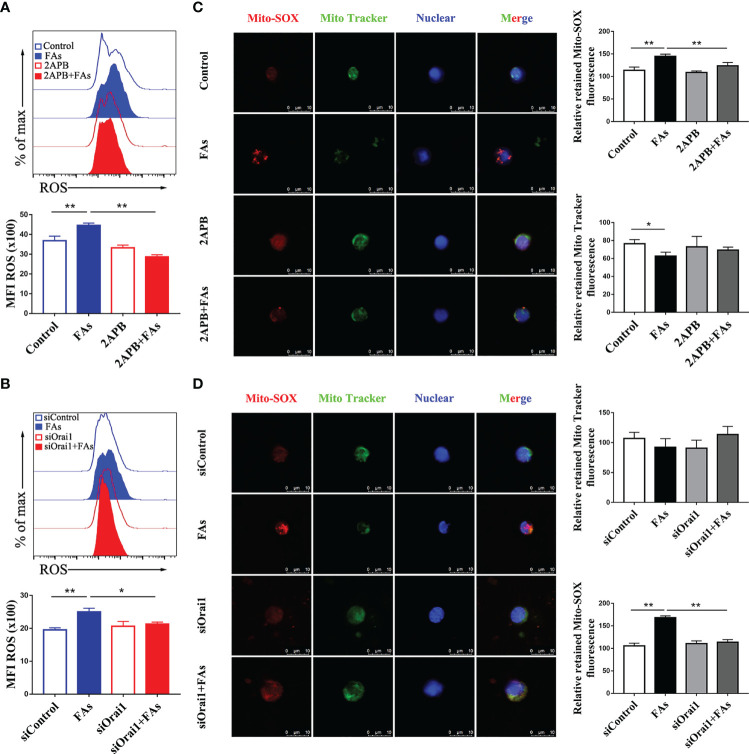
Inhibition or silencing of ORAI1 relieved FAs-induce increased level of intracellular ROS and mitochondrial ROS in CD4^+^ T cells. CD4^+^ T cells were sorted from spleens from 1-d old calves and stimulated with 2APB or siORAI1 before FAs treatment with 12 h **(A)** Staining of intracellular ROS use DCFH-DA in stimulated with 2APB (n = 4 per group). **(B)** Staining of intracellular ROS use DCFH-DA in stimulated with siORAI1 (n = 4 per group). **(C)** Staining of mitochondrial ROS use Mito-SOX in stimulated with 2APB (n = 3 per group; 3 random fields per group; scale bars, 10 μm). **(D)** Staining of mitochondrial ROS use Mito-SOX in stimulated with siORAI1 (n = 3 per group; 3 random fields per group; scale bars, 10 μm). The data of the control were used to normalize other treatments. Comparisons among groups were calculated using a one-way ANOVA with a Duncan correction. The data presented are the mean ± SEM, *P ≤ 0.05, **P ≤ 0.01 indicate differences from control or FAs alone.

We first investigated whether fatty acid stimulated CD4^+^ T cells accumulate Ca^2+^ and ROS, results showed levels of both Ca^2+^ (**Figures 6A, B**) and ROS (**Figures 7A, B**) were greater in CD4^+^ T cells after fatty acid stimulation. Pharmacological inhibition or genetic silencing of ORAI1 also decreased Ca^2+^ (**Figures 6A, B**) and ROS (**Figures 7A, B**) levels in CD4^+^ T cells *in vitro*. In addition, using confocal microscopy with antibodies to Rhod-2 and Mito-SOX, data revealed that Ca^2+^ (**Figures 6C, D**) and ROS (**Figures 7C, D**) accumulated in mitochondria after fatty acid stimulation of CD4^+^ T cells. Accumulation of intracellular Ca^2+^ (**Figures 6C, D**) and ROS (**Figures 7C, D**) was marginal when ORAI1 was silenced or inhibited in CD4^+^ T cells. These results suggested that activation of Orai1 results in an increase in intracellular and mitochondrial ROS and Ca^2+^ levels.

### ORAI1 Regulates Mitochondrial Membrane Permeability and the Abundance of Proteins Associated With Mitochondrial Dynamics in CD4^+^ T Cells

**Figure 8:**
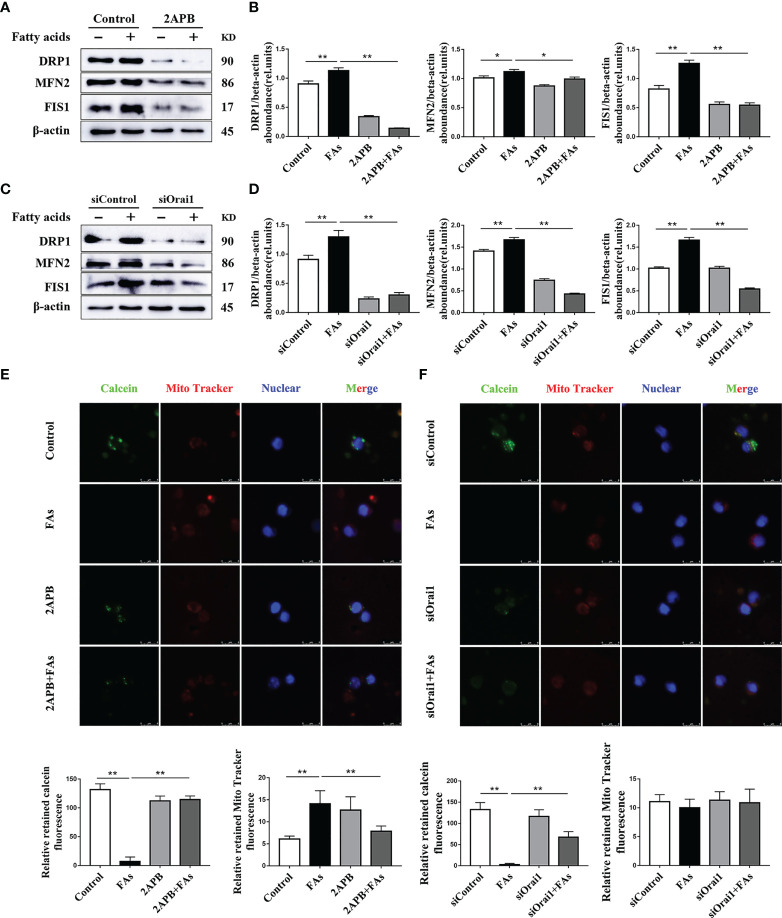
ORAI1 levels affect the expression of mitochondrial dynamics-related protein and autophagy-related protein. CD4^+^ T cells were sorted from spleens from 1-d old calves and stimulated with 2APB or siORAI1 before FAs treatment with 12 h **(A)** Western blot analysis of mitochondrial dynamics-related protein in treatment with 2APB (n = 4 per group). **(B)** Relative abundance of mitochondrial dynamics-related protein in treatment with 2APB (n = 4 per group). **(C)** Western blot analysis of mitochondrial dynamics-related protein in treatment with siORAI1 (n = 4 per group). **(D)** Relative abundance of mitochondrial dynamics-related protein in treatment with siORAI1 (n = 4 per group). **(E)** Opening of mPTP was analyzed in conditions of CD4^+^ T cells treatment with 2APB (n = 3 per group; 3 random fields per group; scale bars, 8 μm). **(F)** Opening of mPTP was analyzed in conditions of CD4^+^ T cells treatment with siORAI1 (n = 3 per group; 3 random fields per group; scale bars, 8 μm). The data of the control were used to normalize other treatments. Comparisons among groups were calculated using a one-way ANOVA with a Duncan correction. The data presented are the mean ± SEM, *P ≤ 0.05, **P ≤ 0.01 indicate differences from control or FAs alone.

To test the role of Orai1 in mitochondrial function in CD4^+^ T cells, we first measured the protein associated with mitochondrial dynamics in CD4^+^ T cells *in vitro*. Fatty acid challenge led to upregulation of protein abundance of targets associated with mitochondrial dynamics (**Figures 8A–D**). Pharmacological inhibition or genetic silencing of ORAI1 attenuated the fatty acid-induced hyperactive division and fusion of mitochondria (**Figures 8A–D**). In addition, compared with control, data indicated that opening of mPTP was greater in cells stimulated with fatty acids (**Figures 8E, F**). Pharmacological inhibition or genetic silencing of ORAI1 attenuated the induction of hyperactive division and fusion of mitochondria (**Figures 8A–D**), and over responsiveness of mPTP (**Figures 8E, F**). These data suggested that the mitochondrial dynamics decreased and opening of mPTP abrogated in the absence of Orai1. These data suggested decreased Orai1 protection fatty acid induced mitochondrial damage.

### ORAI1 Is Involved in Regulating Accumulation of Mitophagy Intermediates

**Figure 9:**
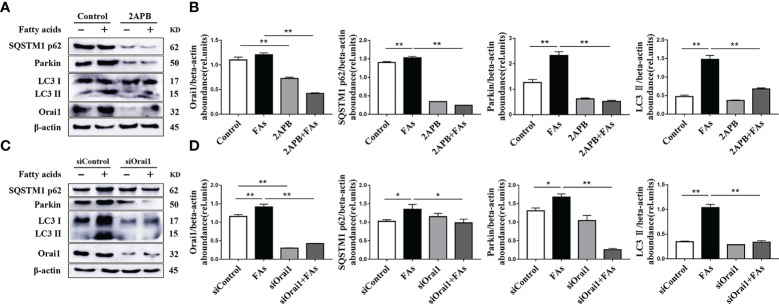
ORAI1 levels affect the expression of autophagy-related protein. CD4^+^ T cells were sorted from spleens from 1-d old calves and stimulated with 2APB or siORAI1 before FAs treatment with 12 h **(A)** Western blot analysis of autophagy-related protein in treatment with 2APB (n = 4 per group). **(B)** Relative abundance of autophagy-related protein in treatment with 2APB (n = 4 per group). **(C)** Western blot analysis of autophagy-related protein in treatment with siORAI1 (n = 4 per group). **(D)** Relative abundance of autophagy-related protein in treatment with siORAI1 (n = 4 per group). The data of the control were used to normalize other treatments. Comparisons among groups were calculated using a one-way ANOVA with a Duncan correction. The data presented are the mean ± SEM, *P ≤ 0.05, **P ≤ 0.01 indicate differences from control or FAs alone.

We sought to understand mitophagy mechanism under fatty acid stimulate conditions in CD4^+^ T cells. To this end, the levels of mitophagy-related proteins were analyzed. Abundance of mitophagy-related proteins such as LC3, p62 and Parkin was upregulated after fatty acid challenge (**Figures 9A–D**), suggesting the initiation of mitophagy. However, upregulation of p62 protein abundance suggested that mitophagy intermediates accumulated during fatty acid challenge (**Figures 9A–D**), along with abundance of ORAI1 (**Figures 9A–D**). To test the direct role of ORAI1 on mitophagy, directly test this idea, ORAI1 in CD4^+^ T cells was inhibited pharmacologically or silenced prior to fatty acid stimulation. Results indicated that pharmacological inhibition or genetic silencing of ORAI1 attenuated the increase in mitophagy-related proteins observed after fatty acid challenge (**Figures 9A–D**). These data suggested that the additional inhibiting or silencing of Orai1 may rescue mitophagy damage caused by fatty acid stimulation in CD4^+^ T cells.

### Inhibition of ORAI1 Attenuates STAT3-Driven Th17 Cell Content

**Figure 10:**
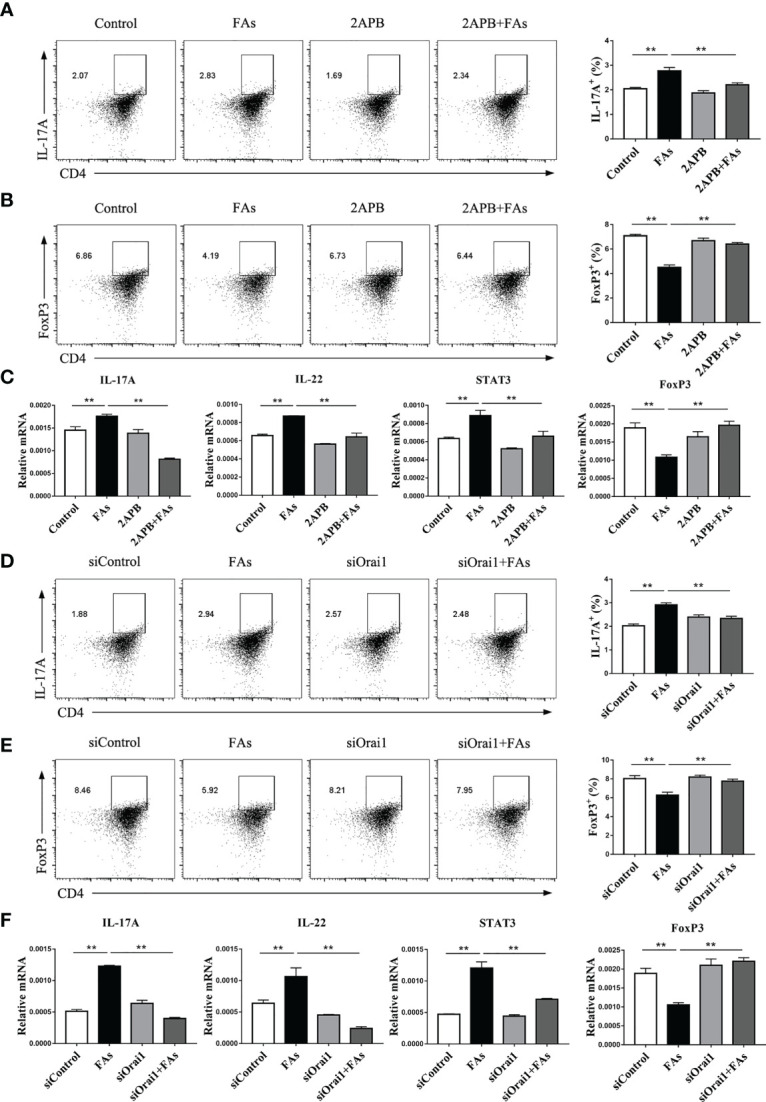
Inhibiting or silencing ORAI1 affects FAs-induced expression of IL-17 and Foxp3 during differentiation of Th17 and Treg cells. CD4^+^ T cells were sorted from spleens from 1-d old calves and stimulated with 2APB or siORAI1 before FAs treatment with 12 h **(A)** Populations of IL-17 cytokine-expressing CD4 T cells in treatment with 2APB (n = 3 per group). **(B)** Populations of FoxP3 expressing CD4 T cells in treatment with 2APB (n = 3 per group). **(C)** Relative mRNA expression of IL-17, STAT3, IL-22 and FoxP3 in treatment with 2APB (n = 4 per group). **(D)** Populations of IL-17 cytokine-expressing CD4 T cells in treatment with siORAI1 (n = 3 per group). **(E)** Populations of FoxP3 expressing CD4 T cells in treatment with siORAI1 (n = 3 per group). **(F)** Relative mRNA expression of IL-17, STAT3, IL-22 and FoxP3 in treatment with siORAI1 (n = 4 per group). The data of the control were used to normalize other treatments. Comparisons among groups were calculated using a one-way ANOVA with a Duncan correction. The data presented are the mean ± SEM, **P ≤ 0.01 indicate differences from control or FAs alone.

In order to assess the role of Orai1 in controlling CD4^+^ T cells differentiation after FAs stimulation, we first directly tested proportion of Th17 cells under fatty acid stimulate conditions *in vitro*. The proportion of Th17 cells increased after fatty acid stimulation (**Figures 10A, D**), but inhibition or silencing of ORAI1 in CD4^+^ T cells under fatty acid challenge reversed this effect (**Figures 10A, D**). Since abundance of STAT3 results in the expansion of Th17 cells (27), we next examined the levels of this protein in CD4^+^ T cells. Fatty acid stimulation upregulated abundance of STAT3 (**Figures 10C, F**), but the level of STAT3 decreased after treatment of CD4^+^ T cells with inhibitor or silencing of ORAI1 (**Figures 10C, F**). Furthermore, pharmacological inhibition or genetic silencing of ORAI1 reduced the abundance of IL-22, a Th17 cell transcriptional signature cytokine (**Figures 10C, F**), suggesting that Orai1 plays an important role in STAT3-driven Th17 cell.

Because the degree of Th17/Treg imbalance has an important role in the context of inflammation, it was important to examine the proportion of Treg cells. Results indicated that fatty acid stimulation decreased the proportion of Treg cells (**Figures 10B, E**), but pharmacological inhibition or genetic silencing of ORAI1 attenuated this effect (**Figures 10B, E**).

### ROS Are Critical Regulators of Mitochondrial Function and Mitophagy in CD4^+^ T Cells Challenged With Fatty Acids

**Figure 11:**
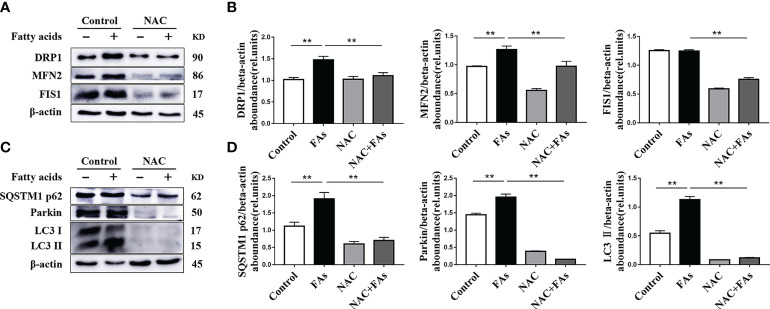
ROS levels affect the expression of mitochondrial dynamics-related protein and autophagy-related protein. CD4^+^ T cells were sorted from spleens from 1-d old calves and stimulated with NAC (10 mM) before FAs treatment with 12 h **(A)** Western blot analysis of mitochondrial dynamics-related protein in CD4^+^ T cells (n = 4 per group). **(B)** Relative abundance of mitochondrial dynamics-related protein (n = 4 per group). **(C)** Western blot analysis of autophagy-related protein in CD4^+^ T cells (n = 4 per group). **(D)** Relative abundance of autophagy-related protein (n = 4 per group). The data of the control were used to normalize other treatments. Comparisons among groups were calculated using a one-way ANOVA with a Duncan correction. The data presented are the mean ± SEM, **P ≤ 0.01 indicate differences from control or NAC alone.

To test the influence of high levels of ROS on CD4^+^ T cell mitochondrial function as well as mitophagy initiation, the ROS scavenger NAC was used to treat CD4^+^ T cells from splenocytes of calves. Compared with fatty acid challenge alone, NAC downregulated abundance of proteins associated with mitochondrial dynamics in cells challenged with fatty acids (**Figures 11A, B**). Furthermore, NAC decreased the fatty acid-induced mitophagy (**Figures 11C, D**). These data suggest that high levels of ROS induce mitochondrial function and mitophagy disorder.

## Discussion

The present studies identified a critical role for ORAI1 on Th17 cell differentiation and pathogenicity through the control of mitochondrial function under a metabolic disorder characterized by elevated concentrations of fatty acids in the circulation. Although mammals experience catabolism of adipose depots to various degrees after parturition (28), high concentrations of fatty acids in the circulation are a hallmark of chronic diseases such as diabetes and obesity. These molecules affect the inflammatory and immune status of the organism (29), and in food producing animals often leads to development of disorders of the reproductive tract and the mammary gland (30).

The innate immune system is a dominant component of the host defense mechanisms, and it is unsurprising that this aspect of immunity has received tremendous attention over the years (31–33). Less appreciated is the role that adaptive immunity plays in the overall context of immune challenges, particularly when innate immune mechanisms fail to eliminate a pathogen or challenge such as high levels of fatty acids. For instance, leukocyte cell-to-cell adhesion was inhibited by high level of fatty acids and increased the susceptibility to infection (34). In the present study, the fact that ketosis was associated with increased levels of pro-inflammatory factors, Th17 cells related factor transcription, and decreased levels of anti-inflammatory factors and Treg cell-related factor transcription factors underscored mechanistic linkages controlling inflammation. The fact that the rate-limiting lipogenic enzyme ACC1 was essential for the inflammation-related pathology of Th17 cells in high-fat-fed mice (35) leads us to speculate that high levels of fatty acids in the circulation enhance peripheral blood Th17 cell differentiation while reducing Treg cell differentiation. Thus, our findings provide evidence for a close relationship between inflammation and CD4^+^ T cells during states that increase circulating concentrations of fatty acids. In this study, we used mixed fatty acids to stimulate calf CD4^+^ T cells. The mixing ratio and concentration are based on previous studies on the fatty acid composition of ketotic cows (36) and other *in vitro* studies (24, 37, 38). But if we use different combinations, the results will be different.

An effective adaptive immune response requires T cells to adapt and function in various microenvironments (39, 40). Since quiescent T-cell energy demands are low, they ensure maximal ATP production in their mitochondria *via* oxidative phosphorylation (OXPHOS) (41, 42). However, upon antigen stimulation, Th cells undergo activation and metabolic reprogramming, switching from a catabolic to an anabolic state in order to provide energy for clonal expansion and effector function (40, 43). Thus, the marked decrease in OXPHOS during ketosis suggested that high concentrations of fatty acids contributed to activation and metabolic reprogramming of lymphocytes.

Activated SOCE provides the bulk of intracellular Ca^2+^ influx after TCR stimulation. It has been suggested that SOCE activates Ca^2+^ dependent enzymes and transcription factors and the nuclear factor of activated T cells (NFAT), which regulate the transcription of several cytokine genes including IL-17A, IL-21, IL-22 and IFNγ (44). A high glucose intake exacerbated autoimmunity and promoted Th17 cell differentiation through increased production of ROS in mice (20). We observed a similar response during ketosis, i.e. high levels of SOCE, ROS and Ca^2+^. Deletion of the electron transport chain (ETC) complex III gene Uqcrfs1 reduced mitochondrial ROS levels and impaired T cell proliferation in mice (45). The role of Ca^2+^ in regulating the structure and function of mitochondria is well known in nonruminants, and mitochondrial Ca^2+^ overload promotes the loss of mitochondrial membrane potential and increases ROS production (46, 47). Thus, we speculate that increased Ca^2+^ content driven by ORAI1 also is responsible for high levels of ROS and subsequent effects on CD4^+^ T cell function, which in turn leads to development of inflammation. Despite inhibition of OXPHOS, a mechanistic explanation for the high ROS levels in CD4^+^ T cells from ketotic cows could be related to upregulation of abundance of ETC complex proteins (including NDUFB8 and UQCRC2). Under that scenario, electron leak would have increased along with ROS production. The end-result of altered mitochondrial metabolism and especially ROS production would further affect inflammatory cytokine secretion and immune cell activation.

Accumulation of ROS leads to oxidative damage, which leads to mitochondrial dysfunction, and induction of autophagy. The latter is characterized by the presence of autophagosomes that engulf aging cytoplasmic organelles and autolysosomes that degrade organelles such as damaged mitochondria. Mitophagy plays a key role in maintaining mitochondrial integrity and is mainly regulated by the PINK1/Parkin signaling pathway (48). It has been reported that Parkin-mediated mitophagy protects metabolic stress-induced mitochondrial damage in endothelial cells (49). Increases in ROS can initiate formation of autophagosomes and degradation of autophagy (50). Furthermore, activation of ROS-mediated PINK1/Parkin signaling causes mitochondrial damage and mitophagy in hepatic stellate cells (51). Thus, the fact that protein abundance of Parkin was upregulated during ketosis and in CD4^+^ T cells treated with fatty acids provided a direct link between these molecules (or indirectly *via* ROS) and induction of mitophagy. Mitophagy is an important process maintaining the stability of mitochondria and homeostasis of the intracellular environment, and works in concert with autophagic flux (52). Thus, data linking both of these essential processes and a metabolic disorder characterized by high levels of fatty acids underscore the biological relevance of the present findings.

Proteins encoded by the LC3 and p62 genes are central for autophagy. The former regulates autophagosome formation and p62 is associated with their degradation (53, 54). The abundance of LC3 protein in T cells is a reliable measure of autophagosome formation and functional activity (55). Thus, the upregulation of LC3 in ketotic cows or fatty acid-challenged CD4^+^ T cells underscored the value of this protein as a biomarker autophagosome formation in the context of metabolic disease. The aggregation of autophagic vesicles is due to the imbalance between autophagosome formation and lysosomal degradation. Alterations in transcription of autophagy-related genes or factors required for autophagosome and lysosome fusion leads to a significant increase in p62-positive aggregates (53). Thus, upregulation of p62 abundance in ketotic cows or fatty acid-challenged CD4^+^ T cells suggested an impairment of mitophagy and accumulation of mitophagy intermediates. We speculate that high circulating concentrations of fatty acids activate autophagosome formation and reduce their degradation in CD4^+^ T cells.

The fact that NAC attenuated induction of ROS production, mitophagy, and mitochondrial separation in activated T cells exposed to high concentrations of fatty acids underscored that activation of mitophagy can occur through changes in ROS production. This is not entirely surprising because a key function of autophagy is to reduce oxidative damage and ROS levels through removal of protein aggregates and damaged organelles such as mitochondria (56). As such, the inability of cells to scavenge excess ROS after impaired mitophagy may be another reason leading to high level of ROS. Although mechanisms of T cell function are likely conserved across mammals, the fact that ruminants do not absorb glucose from the gastrointestinal tract (due to foregut fermentation of dietary carbohydrate) and must rely on gluconeogenesis suggests there could be differences in terms of T cell metabolism that can extend to the control of mitophagy. Further studies are needed to better understand mechanisms in ruminants versus nonruminants.

Although the role of Ca^2+^ in immune cell function is well established, less is known about a role in mitophagy. Previous work in model organisms has suggested that an increase in mitochondrial Ca^2+^ content may influence autophagic pathway (57, 58). The fact that silencing or pharmacological inhibition of ORAI1 in CD4^+^ T cells from calf alleviated fatty acid-induced ROS production and mitochondrial damage suggested a mechanistic link. In that context, the present study has uncovered a novel regulatory pathway responsible for activation of lymphocytes in conditions where circulating concentrations of fatty acids increase, i.e. one that includes intracellular Ca^2+^ signaling and the differentiation of Th17 cells. Mechanistically, the finding that Ca^2+^ can lead to excess ROS production in CD4^+^ T cells links STAT3 transcriptional activity and activation of Th17 (59). This link was strengthened by observations that blockage of ORAI1 by genetic silencing or pharmacological inhibition of ORAI1 attenuated expression of STAT3 and Th17 cell induction *in vitro*. These results suggest that ORAI1 plays an important role in the function of Th17 cells.

**Figure 12:**
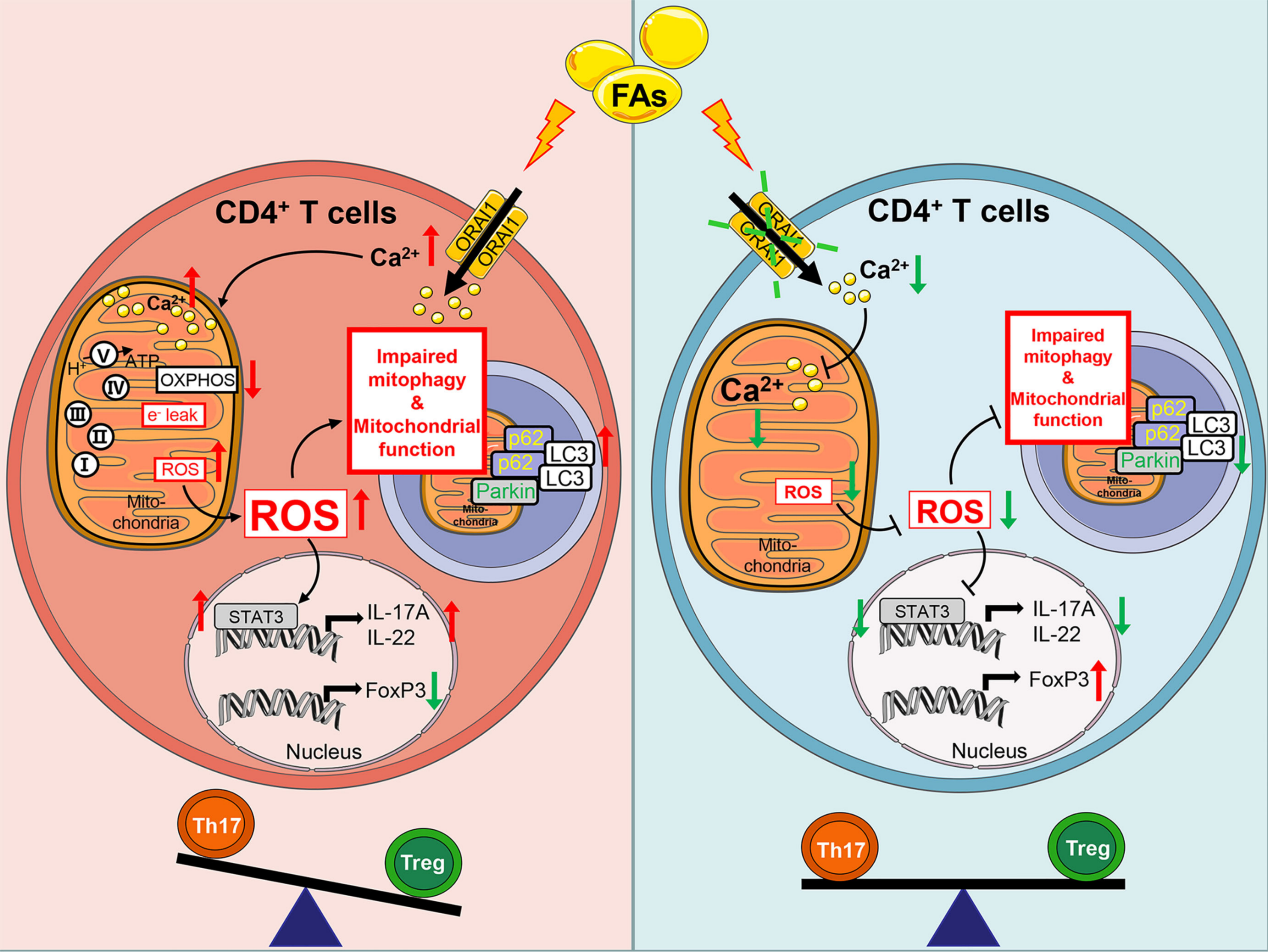
Schematic picture showing Fatty acids impact adaptive immunity negatively through ORAI1 regulated intracellular Ca^2+^, ROS balance, and increased effector functions of Th17 cells in CD4^+^ T cells from cows. Brief in description: Left hand panel. Fatty acids induced ROS production through activated ORAI1. High levels of ROS increased electron leak and mitochondrial membrane permeability to damage mitochondrial function. High levels of ROS increased the protein abundance of Parkin, p62 and LC3 abundance to impair mitophagy and cause accumulation of mitophagy intermediates. Damaged mitochondrial function and impaired mitophagy led to more generation of ROS. ROS increased IL-17A expression and decreased FoxP3 expression by activating STAT3, thus inducing an imbalance between Th17/Treg cells. Right hand panel. Under conditions of fatty acid stimulation, silencing or inhibition of ORAI1 could alleviate the Th17/Treg cells imbalance by downregulating the level of Ca^2+^ and ROS in both mitochondria and cytoplasm.

Based on the data generated, we proposed an empirical model illustrating molecular mechanism (**Figure 12**). Briefly, fatty acids activate ORAI1, increased the level of Ca^2+^ in intracellular which lead to high levels of ROS. Furthermore, ROS through damage mitochondrial function and impaired mitophagy lead to more generation of ROS, which active STAT3 to increased Th17 cells and decreased Treg cells. Therefore, under conditions of fatty acid stimulation, decreased abundance of ORAI1 *via* silencing or pharmacological inhibition could reduce the level of Ca^2+^ and ROS, resulting in a reduction of Th17 cells.

Taken together, previous and current data indicate that the ORAI1-ROS pathway responds to an overload of fatty acids and induces inflammation. Thus, high circulating concentrations of fatty acids can exacerbate CD4^+^ T cell-mediated pathology *via* the induction of ORAI1 underscoring that this protein may represent a therapeutic target to attenuate inflammatory diseases that arise from physiological conditions leading to high concentrations of fatty acids in the circulation such as the postpartum period or chronic diseases such as diabetes and obesity. There is no report in ruminants and we have to admit that lipid metabolism efficiency is related to age in non-ruminant animals (60). Therefore, caution should be taken when trying to extrapolate findings using CD4^+^ T cell from 1-d-old calves to whole-animal CD4^+^ T cell metabolism in periparturient cows. Although *in vivo* data point that ORAI1 signaling was increased in CD4^+^ T cell from ketotic cows and *in vitro* data suggest that ORAI1 signaling could affect CD4^+^ T cells function, the relevance of these data to the transition cow remains to be determined.

In conclusion, ORAI1 through Ca^2+^ regulates the function of mitochondria and consequently determines the pathology of Th17 cells under conditions where concentrations of fatty acids are increased. Thus, ORAI1 could be a potential therapeutic target for the treatment of chronic inflammatory diseases that are associated with sustained Th17 responses in mammalian species.

## Data Availability Statement

The raw data supporting the conclusions of this article will be made available by the authors, without undue reservation.

## Ethics Statement

The animal study was reviewed and approved by Heilongjiang Bayi Agricultural University (Daqing, China). Written informed consent was obtained from the owners for the participation of their animals in this study.

## Author Contributions

Conceived and designed the experiments: BZ and CX. Performed the experiments and analyzed data: ML, WY, YY, JNW, JJW, YH, and SW. Drafted the paper: ML and BZ. Reviewed and edited: JL and QJ. All authors contributed to the article and approved the submitted version.

## Funding

The study was supported by grants from the National Natural Science Foundation of China (32172926, U20A2062) and Heilongjiang Natural Science Foundation (YQ2020C035).

## Conflict of Interest

The authors declare that the research was conducted in the absence of any commercial or financial relationships that could be construed as a potential conflict of interest.

## Publisher’s Note

All claims expressed in this article are solely those of the authors and do not necessarily represent those of their affiliated organizations, or those of the publisher, the editors and the reviewers. Any product that may be evaluated in this article, or claim that may be made by its manufacturer, is not guaranteed or endorsed by the publisher.

## Abbreviations

FA, fatty acids; ROS, reactive oxygen species; SOCE, store-operated Ca^2+^ entry; STIM, stromal interaction molecule; Treg, T regulatory cells; Th, T helper cells; Foxp3, forkhead box protein P3; STAT3, signal transducer and activator of transcription 3; mPTP, mitochondrial permeability transition pore; ER, endoplasmic reticulum; BW, body weight; DIM, days in milk; DMI, dry matter intake; BHBA, β-hydroxybutyrate; PMA, phorbol myristate acetate; OCR, oxygen consumption rate; FCCP, flurorcarbonyl cyanide phenylhydrazone; OXPHOS, oxidative phosphorylation; DRP1, dynamin-related protein 1; MFN2, mitochondrial fusion 2; FIS1, mitochondrial fission 1 protein; SQSTM1, sequestosome-1; PBS, phosphate buffered saline; RT-PCR, reverse transcription polymerase chain reaction; DMSO, Dimethyl Sulfoxide; PVDF, Poly (vinylidene fluoride).
